# Supplementary material for: Hydrophobization of Tobacco Mosaic Virus to Control the Mineralization of Organic Templates
Source: Nanomaterials (Basel). 2019 May 24;9(5):800. doi: 10.3390/nano9050800 (PMC6567237; doi:10.3390/nano9050800)
Supplement: Supplementary file 1 [file nanomaterials-09-00800-s001.pdf]

nanomaterials

Supplementary Materials

Hydrophobization of Tobacco Mosaic Virus to Control the Mineralization of Organic Templates

Petia Atanasova [1,*], Vladimir Atanasov [2], Lisa Wittum [3], Alexander Southan [4], Eunjin Choi [1], Christina Wege [3], Jochen Kerres [2], Sabine Eiben [3] and Joachim Bill [1]

[1] Institute for Materials Science, University of Stuttgart, Heisenbergstr. 3, 70569 Stuttgart, Germany; choi@is.mpg.de (E.C.); bill@imw.uni-stuttgart.de (J.B.)
[2] Institute of Chemical Process Engineering, University of Stuttgart, Böblinger Straße 78, 70199 Stuttgart, Germany; vladimir.atanasov@icvt.uni-stuttgart.de (V.A.); jochen.kerres@icvt.uni-stuttgart.de (J.K.)
[3] Institute of Biomaterials and Biological Systems, University of Stuttgart, Pfaffenwaldring 57, 70569 Stuttgart, Germany; lisawittum1@yahoo.de (L.W.); christina.wege@bio.uni-stuttgart.de (C.W.); sabine.eiben@gmx.de (S.E.)
[4] Institute of Interfacial Process Engineering and Plasma Technology, University of Stuttgart, Pfaffenwaldring 31, 70569 Stuttgart, Germany; alexander.southan@igvp.uni-stuttgart.de
* Correspondence: atanasova@imw.uni-stuttgart.de

Received: 24 April 2019; Accepted: 20 May 2019; Published: date

Transmission measurement of 0.02 g ml^{-1} PPGA in 10 mM SPP buffer solution has shown a lower critical solution temperature of 7.8 °C, while below 4 °C PPGA was completely soluble. The measured concentration was in the same range as used for coupling to TMV.

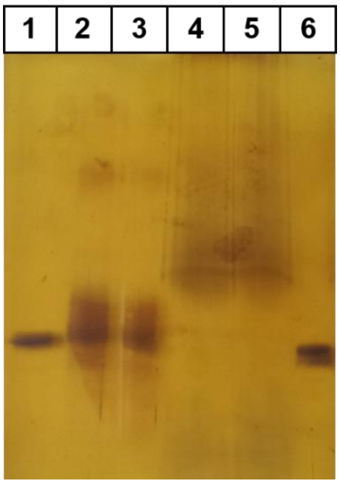

Figure S1. SDS-PAGE of samples containing: (**1**) wt-TMV, (**2**) TMV-ketone, (**3**) TMV-ketone after purification with a PD MiniTrap G-25 column, (**4**) h-TMV, (**5**) h-TMV after purification with a PD MiniTrap G-25 column and (**6**) control experiment including reaction of wt-TMV with PE-b-PEG.

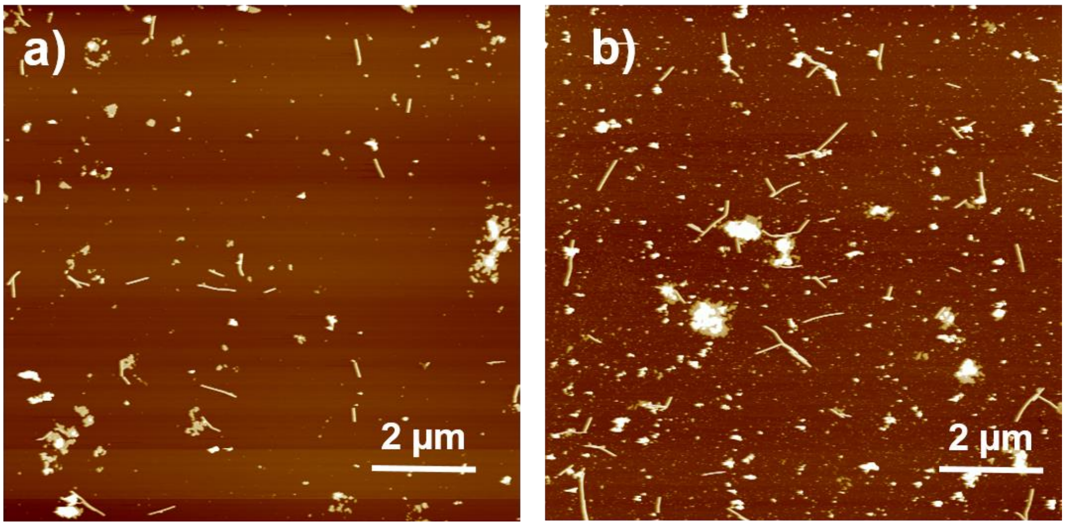

Figure S2. Low magnification AFM height images showing the stability of the h-TMV particles: (a) after 30 min stirring in 100 mM $ZnCl_2$ precursor solution and (b) after 5 min mineralization with ZnS.
